# Skull Base Aneurysmal Bone Cyst Presented with Foramen Jugular Syndrome and Multi-Osseous Involvement

**DOI:** 10.5812/iranjradiol.7952

**Published:** 2012-09-17

**Authors:** Leila Aghaghazvini, Nahid Sedighi, Parisa Karami, Omid Yeganeh

**Affiliations:** 1 Department of Radiology, Shariati Hospital, Tehran University of Medical Sciences, Tehran, Iran; 2 Advanced Diagnostic and Interventional Radiology Research Center (ADIR), Tehran, Iran; 3 Department of Radiology, Medical Imaging Center, Imam Khomeini Hospital, Tehran University of Medical Sciences, Tehran, Iran; 4 Department of Radiology, Tehran University of Medical Sciences, Tehran, Iran; 5 Tehran University of Medical Sciences, Tehran, Iran

**Keywords:** Bone Cysts, Aneurysmal, Petrous Bone, Skull Base, Cranial Fossa,Posterior

## Abstract

Aneurysmal bone cyst (ABC) is an expansile bone lesion that usually involves the long bones. Skull base involvement is rare. Hereby, we describe a 17-year-old man with hoarseness, facial asymmetry, left sided sensorineural hearing loss and left jugular foramen syndrome. CT scan and MRI showed a skull base mass that was confirmed as ABC in histopathology. The case was unusual and interesting due to the clinical presentation of jugular foramen syndrome and radiological findings such as severe enhancement and multiosseous involvement.

## 1. Introduction

Aneurysmal bone cyst (ABC) is a benign osteolytic bony lesion that can be locally destructive. It is usually observed in the first and second decades of life. This expansile bone lesion commonly involves the metaphysis of long tubular bones, posterior elements of the vertebrae and flat bones (1, 2). The skull base is an uncommon location for this tumor with a reported incidence of 3-6% (2, 3). We describe a 17-year-old man with ABC of the skull base which presented with cranial nerve involvement.

## 2. Case Presentation

**Figure 1:**
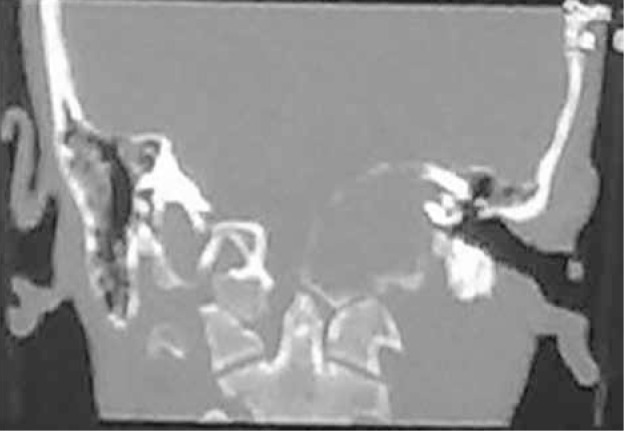
Coronal skull base CT scan Lytic expansile mass on the left side of the hypoglossal foramen extended to jugular foramen and internal auditory canal.

**Figure 2:**
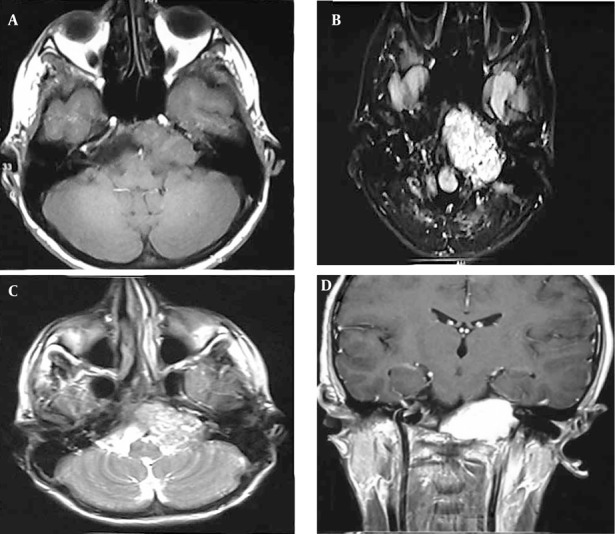
(A-D) Axial and coronal MRI with and without gadolinium: An iso (T1) to heterogeneous iso to high signal (T2, Flair) strongly enhancing lobulated well-defined mass in the left jugular foramen.

A 17-year-old boy presented with a two-year history of hoarseness and facial asymmetry and left sided hearing loss since 5 months ago with no history of trauma or surgery. On physical examination, he had left-sided sensorineural hearing loss, left hemifacial paralysis, deviation of the uvula to the right, loss of left gag reflex and accessory nerve palsy as jugular foramen syndrome. The remainder of head and neck examination was unremarkable. On CT scan, a lytic expansile mass was detected on the left side of foramen magnum and the clivus which involved the jugular foramen and hypoglossal foramen (9th, 10th, 11th and 12th cranial nerves) with extension to the petrous apex and internal auditory canal (7th and 8th nerve complexes) (Figure 1). On MRI, an iso-signal lobulated well-defined mass in T1W sequence containing small high signal foci that was heterogeneous and iso to high signal in T2W and Flair sequences was noted in the jugular foramen and the cerebellopontine angle which showed severe enhancement in post contrast cuts (Figure 2 A-D).

**Figure 3:**
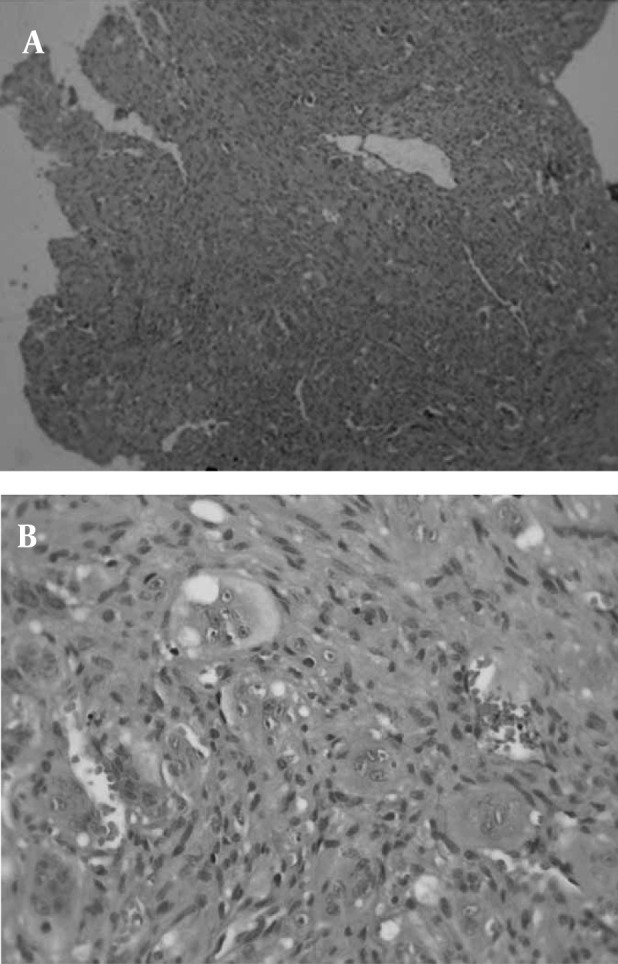
A, Surgical specimen shows cavities surrounded by fibrous connective tissue containing giant cells (hematoxylin-eosin stain, magnification × 10) B, Some scattered giant cells are seen (hematoxylin-eosin stain, magnification × 40)

After resection, histopathology showed multiple blood filled cystic cavities separated by thin fibrous septa and many multinucleated giant cells (Figure 3). Histomorphologic features and immunohistochemical analysis were in favor of aneurysmal bone cyst. Up to six months after resection no recurrence was detected.

## 3. Discussion

Aneurysmal bone cyst is a non-neoplastic osteolytic lesion. This expansile bone lesion contains blood-filled cystic cavities. Of all ABCs, about 2-6% occurs in the skull. The temporal bone, especially the petrous part, is an unusual site of involvement (1-3). In some cases, diagnosis of ABC is difficult because of similar radiologic and pathologic features to other benign and malignant tumors, especially when it occurs in an unusual site like the base of the skull. Clinical presentation of ABCs depends on the site of involvement. As said before, the base of the skull is an unusual site of involvement and most of the temporal bone ABCs occur on the skull vault (4, 5), so in many patients, local swelling with or without pain is the chief complaint. Involvement of adjacent bony and neural structures is not unusual and hearing loss and facial paralysis are seen in some cases (2, 6), but involvement of other cranial nerves is very rare. In our case, because of the special location, multiple cranial nerve involvement was present (7th to 12th cranial nerve palsy) with signs of foramen jugular syndrome and hearing loss. To our knowledge, the skull base ABC with jugular foramen syndrome presentation has not been reported so far. The usual plain skull radiographic abnormalities of ABC are expansile lytic lesions occasionally with thin trabeculation and in some ones, the characteristic pattern of soapbubble has been described (2, 6). In our case, no plain radiography was indicated. Computed tomography shows more details in comparison to the plain radiograph, such as the size of the lesion and its extension. In most cases there is an expansile lytic lesion with fine internal septation and a well-defined thin margin. Following intravenous administration of iodinated contrast, the margin of the lesion and internal septation both enhance strongly. The classic pattern of fluid-fluid level is seen in some patients and represents the sedimentation of RBCs in hemorrhagic cavities (2, 5), but it is not a specific pattern and could be seen in telangiectatic osteosarcoma,chondroblastoma, fibrous dysplasia, simple bone cyst, recurrent malignant fibrous histiocytoma and classical osteosarcoma. However, in association with other signs it could be a good clue (7, 8). On MRI of skull bone ABC, usually a well-defined expansile mass is seen surrounded by a hypointense rim. In most cases, the lesion has an internal septation that divides it to small cavities and sometimes the wall of these cavities has diverticulumlike projections (2, 9). MRI also shows fluid levels, especially in T1 weighted images. After gadolinium injection, intense contrast enhancement is seen at the peripheral capsule and the internal septations. In our case, CT scan showed a lytic expansile mass on the left side of foramen magnum, clivus and the jugular foramen with extension to the petrous apex and internal auditory canal (Figure 1). On MR imaging of this case an iso to heterogeneous (T1), iso to high-signal (T2, Flair) strongly enhancing lobulated well-defined mass in the left jugular foramen and cerebellopontine angle was seen that was somehow different from typical MRI findings of these tumors. As mentioned, according to MR findings, primary tumors including glomus jugular tumors, schwannomas, meningiomas and peripheral primitive neuroectodermal tumors, based on CT scan and MRI findings, giant cell tumor and based on the patient’s age, ABC were our differential diagnosis. Total excision is the perfect treatment of ABC, which could be curative. However, in some patients treatment of skull base involvement with total excision is difficult and radiotherapy has been recommended (6, 10). Khaldi et al. introduced intralesional injection of calcitonin after partial resection as the successful treatment in cranial ABC as a new treatment (11) After definitive histopathological diagnosis, our patient underwent total excision and no recurrence was found after six months. In summary, although ABC is a benign bone lesion, in the current case its specific location leads to multiosseous involvement with a very unusual presentation of foramen jugular syndrome affecting adjacent cranial nerves which has not been reported before.

## References

[1] Resnick D, Resnick D (2003). Tumor and tumor-like lesion of bone: imaging and pathology of specific lesions. Bone & joint imaging.

[2] Sabatini PR, Horenstein MG, Oliveri CV, Gacek RR (2005). Aneurysmal bone cyst of the temporal bone associated with reversible hemifacial paralysis. Am J Otolaryngol.

[3] Sayama CM, MacDonald JD (2010). Aneurysmal bone cyst of the petrous bone: case presentation and review of the literature. Pediatr Neurosurg.

[4] Muzumdar DP, Goel A, Mistry R, Gujral S, Fattepurkar S (2004). Postoperative cerebellar herniation in a large intrapetrous aneurysmal bone cyst. J Clin Neurosci.

[5] Sheikh BY, Kanaan I, Alwatban J, Enazi A, Patay Z (1999). Aneurysmal bone cyst involving the skull base: report of three cases. Skull Base Surg.

[6] Cakirer S, Basak M, Celebi I, Kabukcuoglu F, Erdem Y (2003). Aneurysmal bone cyst of the temporal bone. Curr Probl Diagn Radiol.

[7] Munk PL, Helms CA, Holt RG, Johnston J, Steinbach L, Neumann C (1989). MR imaging of aneurysmal bone cysts. AJR Am J Roentgenol.

[8] Tsai JC, Dalinka MK, Fallon MD, Zlatkin MB, Kressel HY (1990). Fluid-fluid level: a nonspecific finding in tumors of bone and soft tissue. Radiology.

[9] Shah GV, Doctor MR, Shah PS (1995). Aneurysmal bone cyst of the temporal bone: MR findings. AJNR Am J Neuroradiol.

[10] Purohit A, Chopra S, Sinha VD, Dharker SR (2002). Aneurysmal bone cyst of the temporal bone: a case report. Neurol India.

[11] Khaldi M, Ben Hamouda K, Moussa M, Megdiche H, Boubaker A, Marrakchi M (2006). [Aneurysmal bone cyst of the cranial base treated by partial resection and calcitonin injection. A case report].. Neurochirurgie.

